# RETRACTION: Evaluation of the Genotoxic Potential against H_2_O_2_‐Radical‐Mediated DNA Damage and Acute Oral Toxicity of Standardized Extract of *Polyalthia longifolia* Leaf

**DOI:** 10.1155/ecam/9780452

**Published:** 2026-06-26

**Authors:** Evidence-Based Complementary and Alternative Medicine

RETRACTION: S. L. Jothy, Y. Chen, J. R. Kanwar, and S. Sasidharan, “Evaluation of the Genotoxic Potential against H_2_O_2_‐Radical‐Mediated DNA Damage and Acute Oral Toxicity of Standardized Extract of *Polyalthia longifolia* Leaf,” *Evidence-Based Complementary and Alternative Medicine* 2013, no. 1 (2013): 1–13, https://doi.org/10.1155/2013/925380.

The above article, published online on 24 June 2013 in Wiley Online Library (https://wileyonlinelibrary.com), and its correction (https://onlinelibrary.wiley.com/doi/10.1155/2021/9836793) has been retracted by John Wiley & Sons Ltd. The retraction has been agreed due to figure concerns. Following the initial correction of Figure 3, additional concerns were identified on PubPeer [1] and investigated. Specifically:

• Figure 3(a1) and 3(a2) appear to contain overlapping sections despite different experimental conditions
• Figure 3(b1) and 3(b2) appear to be similar to Figure 2(d1) and 2(d2) in [2] despite different experimental conditions

The authors were not able to provide the original figures when requested. The results and conclusions are therefore considered unreliable.

The authors disagree with the retraction.
